# Comparative visual ecophysiology of mid-Atlantic temperate reef fishes

**DOI:** 10.1242/bio.20136825

**Published:** 2013-11-06

**Authors:** Andrij Z. Horodysky, Richard W. Brill, Kendyl C. Crawford, Elizabeth S. Seagroves, Andrea K. Johnson

**Affiliations:** 1 Department of Marine and Environmental Science, Hampton University, Hampton, VA 23664, USA; 2 National Marine Fisheries Service, Northeast Fisheries Science Center, James J. Howard Marine Sciences Laboratory, Sandy Hook, Highlands, NJ 07732, USA; 3 Department of Fisheries Science, Virginia Institute of Marine Sciences, College of William & Mary, Gloucester Point, VA 23062, USA; 4 Department of Natural Resources, University of Maryland Eastern Shore, Princess Anne, MD 21853, USA

**Keywords:** Electroretinography, Fish, Flicker fusion frequency, Spectral sensitivity, Temperate reef, Visual ecology

## Abstract

The absolute light sensitivities, temporal properties, and spectral sensitivities of the visual systems of three mid-Atlantic temperate reef fishes (Atlantic spadefish [Ephippidae: *Chaetodipterus faber*], tautog [Labridae: *Tautoga onitis*], and black sea bass [Serranidae: *Centropristis striata*]) were studied via electroretinography (ERG). Pelagic Atlantic spadefish exhibited higher temporal resolution but a narrower dynamic range than the two more demersal foragers. The higher luminous sensitivities of tautog and black sea bass were similar to other benthic and demersal coastal mid-Atlantic fishes. Flicker fusion frequency experiments revealed significant interspecific differences at maximum intensities that correlated with lifestyle and habitat. Spectral responses of the three species spanned 400–610 nm, with high likelihood of cone dichromacy providing the basis for color and contrast discrimination. Significant day-night differences in spectral responses were evident in spadefish and black sea bass but not tautog, a labrid with characteristic structure-associated nocturnal torpor. Atlantic spadefish responded to a wider range of wavelengths than did deeper-dwelling tautog or black sea bass. Collectively, these results suggest that temperate reef-associated fishes are well-adapted to their gradient of brighter to dimmer photoclimates, representative of their unique ecologies and life histories. Continuing anthropogenic degradation of water quality in coastal environments, at a pace faster than the evolution of visual systems, may however impede visual foraging and reproductive signaling in temperate reef fishes.

## Introduction

The evolutionary radiation of fishes into a wide range of aquatic habitats with unique photic properties has resulted in a myriad of selective forces on fish visual systems (Levine and MacNichol, 1979; Collin, 1997). Waters with different properties disparately scatter and absorb downwelling light, affecting its spectral bandwidth (color) and intensity (brightness) with depth. Pure natural waters and clear pelagic seas act as monochromators, maximally transmitting short (blue) wavelengths, whereas intermediate (green) wavelengths maximally penetrate coastal waters (Jerlov, 1968). The ambient spectrum in estuarine and fresh waters shifts to longer (yellow-red) wavelengths as increased primary productivity, dissolved organics, and suspended particulates more rapidly attenuate light (Lythgoe, 1975; Lythgoe, 1988). Near-surface waters can vary in irradiance by a daily range of six to nine orders of magnitude depending on the moon phase; scatter and absorption further restrict the spectral bandwidth and intensity of downwelling light with depth (McFarland, 1986; Warrant, 2000).

The structural and functional characteristics of fish visual systems generally reflect the characteristics of aquatic light fields (Guthrie and Muntz, 1993). Species with duplex retinae may using cone cells under photopic (bright) conditions and rod cells during scotopic (dim/dark) conditions to extend visual performance (Lythgoe, 1979; Crescitelli, 1991). However, unavoidable tradeoffs between visual sensitivity and temporal or spatial resolution render optimal visual performance nearly impossible to maintain over the full range of daily optical conditions (Warrant, 1999). As a result, morphological adaptations and physiological performance of teleost eyes vary depending on physical, environmental, and phylogenetic constraints and are thus informative of a species' ecology, lifestyle, and habitat (Levine and MacNichol, 1979; Collin and Marshall, 2003). Comparative methods have provided novel insights into the form–function–environment relationships of the fish eye (Walls, 1942; Levine and MacNichol, 1979; Parkyn and Hawryshyn, 2000; Jokela-Määtä et al., 2007), fish movements and their distributions (McFarland, 1986), mechanisms of communication (Siebeck et al., 2006), predator–prey interactions (Browman et al., 1994; De Robertis et al., 2003), and vulnerability to sampling gear (Buijse et al., 1992; Weissburg and Browman, 2005; Kotwicki et al., 2009). Much research has focused on the properties of fish photoreceptor cells, their pigments, and correlations to the photic properties of habitats (McFarland and Munz, 1975; Dartnall, 1975; Levine and MacNichol, 1979; Bowmaker, 1990; Losey et al., 2003).

Form:function relationships in the visual systems of tropical reef fishes have received fairly rigorous attention in the literature, yet very little is known about their temperate analogues. Coral reef environments are characterized by clear waters and intense solar radiation, resulting in high spectral complexity as different habitats within reef environments have distinct irradiance spectra (McFarland and Munz, 1975; Barry and Hawryshyn, 1999; Marshall et al., 2003a; Marshall et al., 2003b). Within tropical reefs, optical macrohabitats grade from blue waters of the outer reef to progressively greener waters of the middle and inner reef (Myrberg and Fuiman, 2002), with each region further having a multitude of spectrally-distinct optical microhabitats (Marshall, 2000; Marshall et al., 2006). Coral reef fishes thus demonstrate a stunningly diverse array of body colorations and visual pigments, light niches, foraging strategies, and lifestyles (Marshall et al., 2003a; Siebeck et al., 2008). Although both taxonomic representatives and ecological analogues of many groups of coral reef fishes are found on temperate reefs, hardbottom habitats, and manmade offshore structures, surprisingly little is known about the visual function and tasks in fishes that associate with these environments.

Recent comparative investigations of visual ecophysiology in coastal fishes have used corneal electroretinography (ERG) to assess visual function in phylogenetically-related fishes that use different optical microhabitats (Horodysky et al., 2008; Horodysky et al., 2010) and phylogenetically-dissimilar fishes with interacting trophic ecologies and habitat preferences (Horodysky et al., 2010; McComb et al., 2013). We therefore used this same technique to assay the absolute sensitivities, temporal properties, and chromatic sensitivities of three structure-associated temperate reef fishes with dissimilar phylogenies and feeding ecologies. The objective of our study was to investigate the relationship between form, function, and the environment, and to place the visual systems of these three temperate reef teleosts in context of other temperate coastal and tropical marine fishes.

## Materials and Methods

**Fig. 1.**
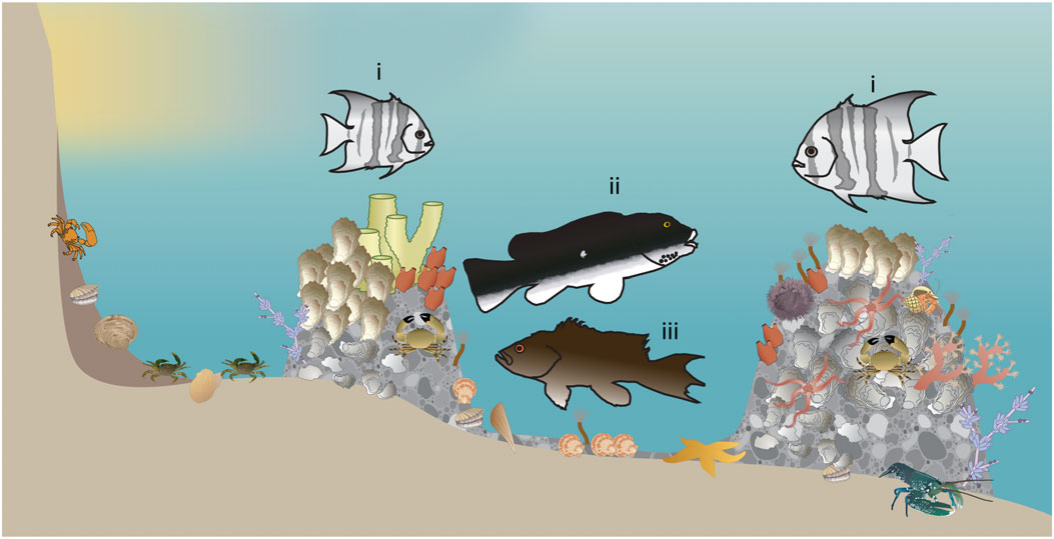
Conceptual diagram of the microhabitat specialization of the three mid-Atlantic temperate reef fishes examined in this study. Spadefish (i) are predators of cnidarians, bivalves, and small crustaceans, often schooling in large numbers above hardbottom habitats, shipwrecks, and marine construction platforms (Hayse, 1990). Tautog (ii) are predators of mollusks and crustaceans, demonstrate strong association with natural and manmade structures, and undertake seasonal inshore-offshore movements induced by temperature changes (Olla et al., 1974; Olla et al., 1980; Auster, 1989; Clark et al., 2006). Black sea bass (iii) are structure-associated predators of a myriad of crustaceans, bivalves, and small to medium-sized fishes (Steimle and Figley, 1996). Juveniles of these three species use estuarine waters as nursery and foraging grounds. Invertebrate symbols are courtesy of the Integration and Application Network, University of Maryland Center for Environmental Science (http://ian.umces.edu/symbols).

Experimental and animal care protocols were approved by the College of William & Mary's Institutional Animal Care and Use Committee and followed all relevant laws of the United States. Atlantic spadefish (*Chaetodipterus faber* Broussonet 1782) were obtained from the Virginia Institute of Marine Science's hatchery program; tautog (*Tautoga onitis* Linnaeus 1758) and black sea bass (*Centropristis striata* Linnaeus 1758) were captured in the wild by standard hook and line fishing gear (Fig. 1; Table 1). Animals were maintained in recirculating 1855 L aquaria on natural ambient photoperiods at 18°C ± 2°C. Spadefish were fed commercial pelleted feed (AquaMax Grower 600, Purina Mills, Gray Summit, MO, USA). Tautog and sea bass were fed a combination of frozen Atlantic menhaden (*Brevoortia tyrannus*), grass shrimp (*Palaemonetes* sp.), and assorted bivalves.

**Table 1.**
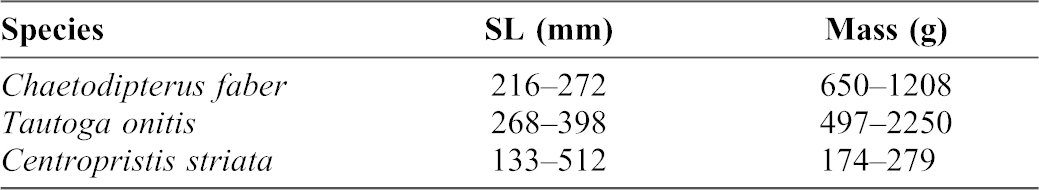
Species, standard length (SL), and mass of the three Mid-Atlantic temperate reef fishes investigated in this study.

Fish were netted from holding tanks, given an anaesthetic dose of ketamine hydrochloride (30 mg kg^−1^, Butler Animal Health, Middletown, PA, USA) and then immobilized with the neuromuscular blocking drug gallamine triethiodide (Flaxedil; 10 mg kg^−1^, Sigma, St. Louis, MO, USA), both delivered intramuscularly. Subjects were then rapidly transferred into a light-tight enclosure (maintained in a darkened room) and placed in a rectangular 800 × 325 × 180 mm Plexiglas tank with only a small portion of the head and eye remaining above the water to receive the light stimulus. Subjects were ventilated with filtered and oxygen-saturated sea water (0.5–1 L min^−1^) that was temperature-controlled (20 ± 2°C) to minimize the potential confounding effects of temperature on ERG recordings (Saszik and Bilotta, 1999; Fritsches et al., 2005). Fish were dark adapted for at least 45 min prior to any measurements (following Horodysky et al., 2008). Drugs were readministered during experiments as required.

Experiments were conducted during both day and night hours (defined following local ambient photoperiods) to determine any circadian rhythms in visual responses (McMahon and Barlow, 1992; Cahill and Hasegawa, 1997; Mangel, 2001). At the conclusion of each experiment, fishes were euthanized via a massive overdose (>300 mg kg^−1^) of sodium pentobarbital (Beuthanasia-D, Schering-Plough Animal Health Corp., Union, NJ, USA) injected intramuscularly.

### Electroretinography (ERG)

Whole-animal corneal ERGs were used to assess the absolute sensitivities, temporal properties, and spectral sensitivities of fish visual systems. Teflon-coated silver-silver chloride electrodes were used for recording responses. The active electrode was placed on the corneal surface and a reference electrode was placed subdermally in the dorsal musculature. ERG recordings and stimulus presentations were controlled using software developed within the LabVIEW system (National Instruments, Austin, TX, USA) by Eric Warrant (University of Lund, Lund, Sweden).

Absolute luminous sensitivities were assessed via intensity-response (V/logI) experiments as described in Horodysky et al. (Horodysky et al., 2008). Briefly, up to six orders of magnitude of stimulus intensity were presented to subjects using combinations of Kodak Wratten 1.0 and 2.0 neutral density filters (Eastman Kodak Co., Rochester, NY, USA) and a white LED light source (Advanced Illumination SL-2420-WHI) with a working range of roughly three log_10_ units and a maximum output intensity of 1585 cd m^−2^. Light intensities were calibrated with a research radiometer (model IL 1700, International Light, Inc., Newburyport, MA). V/logI experiments progressed in 0.2 log unit steps from subthreshold to saturation intensity levels. At each intensity step, ERG b-waves were recorded from a train of five 200 ms flashes, each separated by 200 ms rest periods. This process was repeated five times and normalized to the maximum voltage response (V_max_). Mean V/logI curves for each species averaged the V/logI curves of individuals of that species. Interspecific comparisons of relative luminous sensitivity were made at stimulus irradiances eliciting 50% of V_max_ (referred to as K_50_). Dynamic ranges, defined as the log_10_ irradiance range between the limits of 5–95% V_max_ (*sensu* Frank, 2003), were calculated separately for day and night experiments.

The temporal resolution of sciaenid visual systems was assessed via flicker fusion frequency (FFF) experiments using the white light LED source above following Fritsches et al. (Fritsches et al., 2005). Sinusoidally-modulated white light stimuli ranging in frequency from 1 Hz (0 log units) to 100 Hz (2.0 log units) were presented to subjects in 0.2 log unit frequency steps, repeated five times at each frequency, and averaged for each subject. Light stimuli were presented for 5 s, followed by 5 s of darkness. Seven total FFF experiments were conducted for each subject: one at 25% (I_25_) of maximum stimulus intensity (I_max_) determined from the V/logI curve, and one in each log_10_ step interval over six orders of magnitude of light intensity. A subject's FFF threshold at a given intensity was determined by analyzing the power spectrum of the averaged responses from 1–100 Hz and comparing the power of the subject's response frequency (signal) to the power of a neighboring range of noise frequencies (Horodysky et al., 2010). Diel and interspecific comparisons were conducted on the FFF data at I_max_ and I_25_. The FFF at I_25_ has been used as a very general proxy for ambient photopic light intensity (Horodysky et al., 2008; Horodysky et al., 2010) for use in comparing across species, and the FFF at I_max_ is the maximum flicker fusion frequency attainable by the visual system of a given species (Horodysky et al., 2008).

Spectral sensitivity experiments were conducted to assess the ability of the visual systems of temperate reef fishes to respond to colored light stimuli that covered the spectral range from UV (300 nm) to the far red (650 nm) in 10 nm steps (following Horodysky et al., 2008). The output of a Cermax Xenon fiberoptic light source (ILC Technology, Sunnyvale, CA, USA) was controlled by a CM110 monochromator, collimated, and passed through each of two AB301 filter wheels containing quartz neutral density filters (CVI Laser Spectral Products, Albuquerque, NM, USA) which together allowed the attenuation of light from 0 to 5 log units in 0.2 log unit steps. The LabVIEW program delivered stimuli by controlling a Uniblitz LS6 electronic shutter (Vincent Associates, Rochester, NY, USA) using the analog and digital output of the DAQ card and the computer's serial RS232 interface. Five single 40 ms stimulus flashes were presented through a 1 cm diameter quartz light guide placed within approximately 5 cm of a subject's eye at each experimental wavelength, each followed by 6 s of darkness. The amplitudes of ERG responses were recorded and averaged to form raw spectral response curves for each individual. A spectral V/logI recording was subsequently conducted for each subject at the wavelength (λ_max_) that generated its maximum ERG response (V_max_) to facilitate the subsequent calculation of the subject's spectral sensitivity curve at equal quantal light intensities at each wavelength. Spectral V/logI experiments exposed the subject to five individual monochromatic (50% bandwidth 5 nm) flashes of 200 ms duration at each intensity, increasing in 0.2 log unit increments over five orders of magnitude. Spectral response voltages were transformed to spectral sensitivities for each subject by converting the former to equivalent intensities and expressing on a percentage scale (100% indicating maximum sensitivity), following Eqn 1:


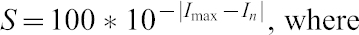
(1)

S = spectral sensitivity

I_max_ = intensity at maximum response voltage

I_n_ = intensity at response voltage *n*

Finally, spectral sensitivity curves for each species were averaged from the sensitivity curves of all subjects. These were subsequently normalized to each species' maximum resulting value so that all species' maximum sensitivity equaled 100%.

## Data analyses

### V/logI and FFF

Temperate reef fish V/logI and FFF data were analyzed separately using two-way repeated measures ANOVAs with Tukey's post hoc comparisons to assess whether ERG responses varied among the three species and between photoperiods. All statistical analyses were conducted using SAS v9 (SAS Institute, Cary, NC, USA). A general model for these analyses is given in Eqn 2:


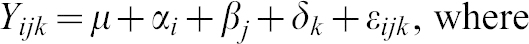
(2)

Y_ijk_ = value of the response variable (response) for the *i*^th^ species, *j*^th^ diel period, and the *k*^th^ level of their interaction

μ = overall mean of threshold for all combinations of species and diel periods

α*_i_* = species (fixed factor)

β*_j_* = diel period (fixed factor)

δ*_k_* = species:diel interaction

ε*_ijk_* = random error term associated with the observation at each combination of the i^th^ species, the j^th^ diel period, and k^th^ level of their interaction.

### Spectral sensitivity

Intraspecific diel differences in spectral sensitivity curves were assessed by subtracting the day and night curves and calculating confidence intervals (CI) of the resulting difference curve (following Horodysky et al., 2010). Positive values correspond to increased day sensitivity; negative values indicate increased nocturnal sensitivity. Significant differences in spectral sensitivity occurred where the mean ± CI of difference curves did not encompass zero.

To form hypotheses regarding the number and spectral distribution of pigments potentially contributing to spectral ERG responses, we fitted the SSH (Stavenga et al., 1993) and GFRKD (Govardovskii et al., 2000) vitamin A1 rhodopsin absorbance templates separately to the photopic spectral sensitivity data (Horodysky et al., 2008; Horodysky et al., 2010). As none of the species responded to ultraviolet wavelengths, we considered scenarios of 1–3 α-band rhodopsins with no β-bands on any pigment. For a given species, condition and template, models of summed curves were created by adding the products of pigment-specific templates and their respective weighting factors. Estimates of the unknown model parameters (λ_max_ values and their respective weighting proportions) were derived by fitting the summed curves to the ERG data using maximum likelihood.

For each species, we objectively selected the appropriate template (SSH or GFRKD) and number of contributing pigments using an Information Theoretic approach (Burnham and Anderson, 2002) following Akaike's Information Criterion (AIC) (Eqn 3):


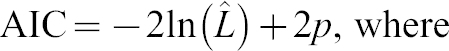
(3)

AIC: Akaike's Information Criterion


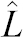
: the estimated value of the likelihood function at its maximum

*p*: number of estimated parameters

This technique balances model complexity and parsimony in selecting the conditions that best explain the underlying data. All parameter optimization, template fitting, and model selection was conducted using the software package *R* version 2.12.1 (R Development Core Team 2008).

## Results

**Fig. 2.**
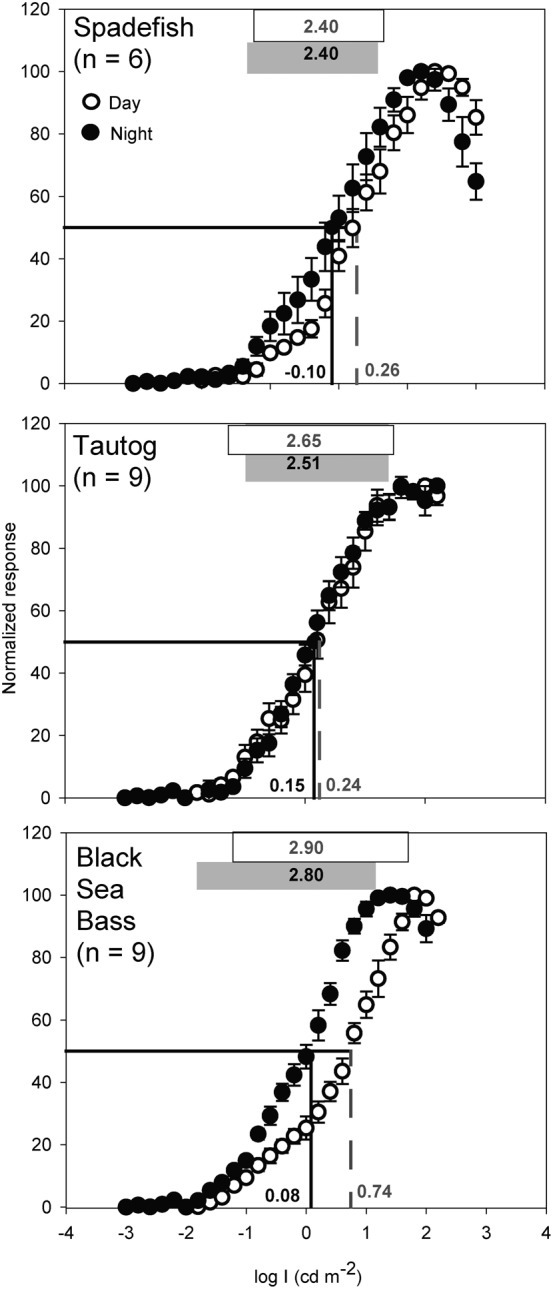
Intensity-response electroretinograms (ERGs) of Atlantic spadefish, tautog, and black sea bass. Each species' intensity response curve is an average of six to nine individuals. Responses were normalized to the maximal response voltage (V_max_) for each individual. Boxes at the top represent each species' dynamic range (5–95% V_max_), numbers at the top indicate its breadth (in log units). Dashed drop lines and adjacent numbers indicate K_50_ points (illumination at 50% V_max_). Open symbols, white boxes, and grey text represent day experiments, filled symbols, shaded boxes, and black text represent night experiments. Light intensities are in log candela m^−2^. Error bars are ± 1 SE.

White-light evoked ERG b-wave responses of the three temperate reef fishes increased non-monotonically with stimulus intensity to maximum amplitudes (V_max_) of 50–800 µV, then decreased at intensities above V_max_ (Fig. 2), presumably due to photoreceptor saturation and a lack of pigment regeneration. The K_50_ values of V/logI curves varied significantly between diel periods (F_1,19_ = 14.27, *P*<0.002) but not among species (F_2,19_ = 2.32, *P*>0.05). Interaction terms were not significant. Tukey's post-hoc comparisons revealed that the mean photopic K_50_ values of black sea bass were significantly right-shifted (0.5 log units, *P*<0.05) relative to Atlantic spadefish and tautog, indicating reduced sensitivity to dim light during daylight hours in the former. Mean photopic dynamic ranges of the three species, defined as 5–95% of V_max_, varied between 2.4–2.9 log units and scotopic dynamic ranges between 2.4–2.8 log units. Dynamic ranges varied significantly among the species (F_2,19_ = 6.71, *P*<0.007), but not diel periods (F_2,19_ = 0.42, *P*>0.05); interaction terms were not significant. Black sea bass and tautog had wider dynamic ranges than spadefish.

**Fig. 3.**
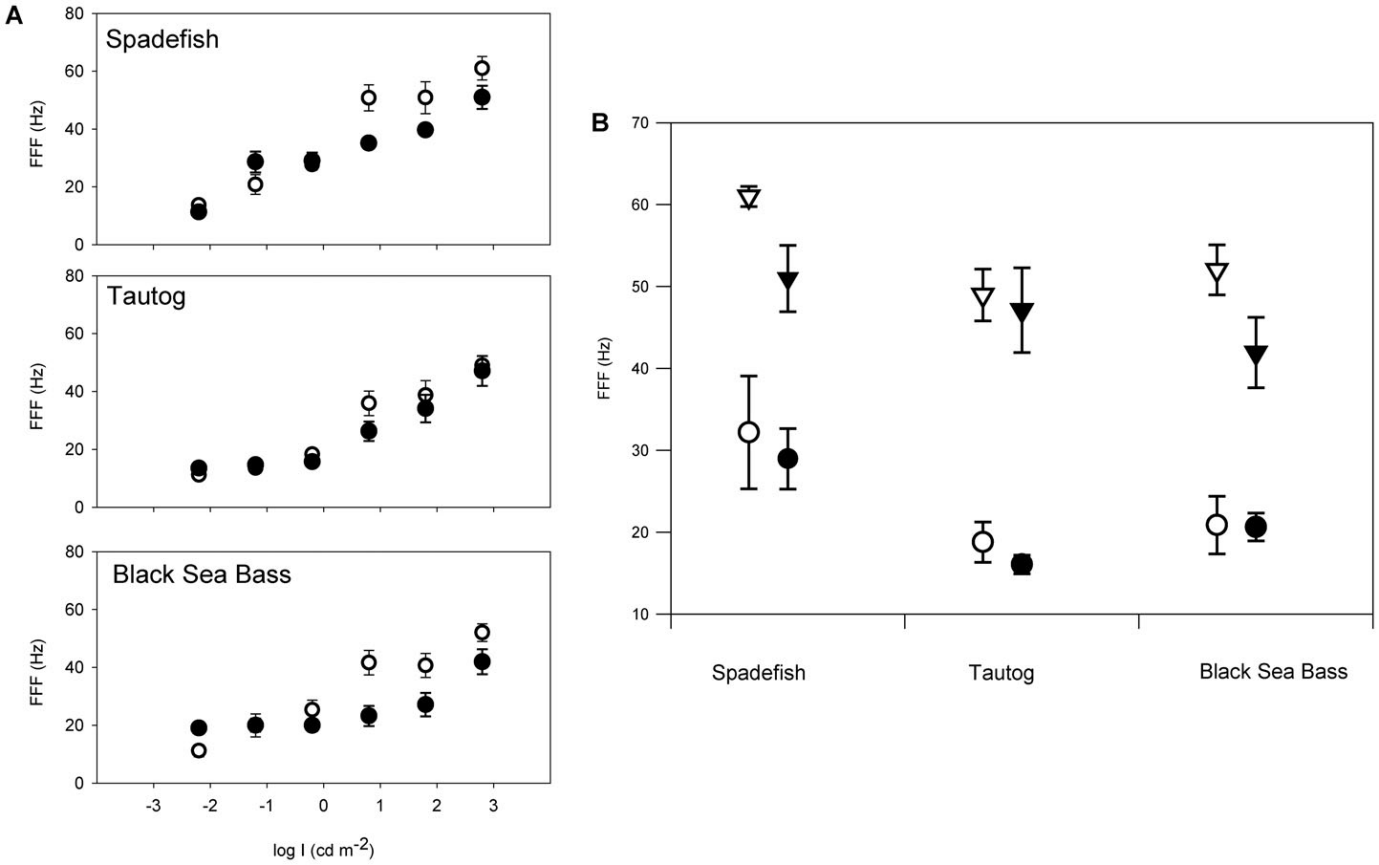
The relationship between light intensity and flicker fusion frequency (FFF) for Atlantic spadefish, tautog, and black sea bass. Open symbols represent day experiments, filled symbols represent night experiments. Error bars are ± 1 SE. A. FFF over six orders of magnitude of light intensity for the three temperate reef fishes. B. Mean flicker fusion frequency (FFF) values for the temperate reef fishes at I_25_ (light levels 25% of I_max_; circles) and I_max_ (maximum stimulus intensity; triangles). We considered I_25_ to be a proxy for ambient environmental light intensity (*sensu* Horodysky et al., 2008).

The FFF values of temperate reef fishes (Fig. 3A,B) varied among species (F_2,19_ = 5.07, *P*<0.02), with spadefish having significantly higher photopic values than tautog and black sea bass. FFF increased with increasing intensity (i.e., greater at I_max_ than I_25_; F_1,62_ = 142.95, *P*<0.0001). However, there was no significant nocturnal difference among FFF values between diel periods (F_1,62.8_ = 0.11, *P*>0.05). Interaction terms were not significant.

**Fig. 4.**
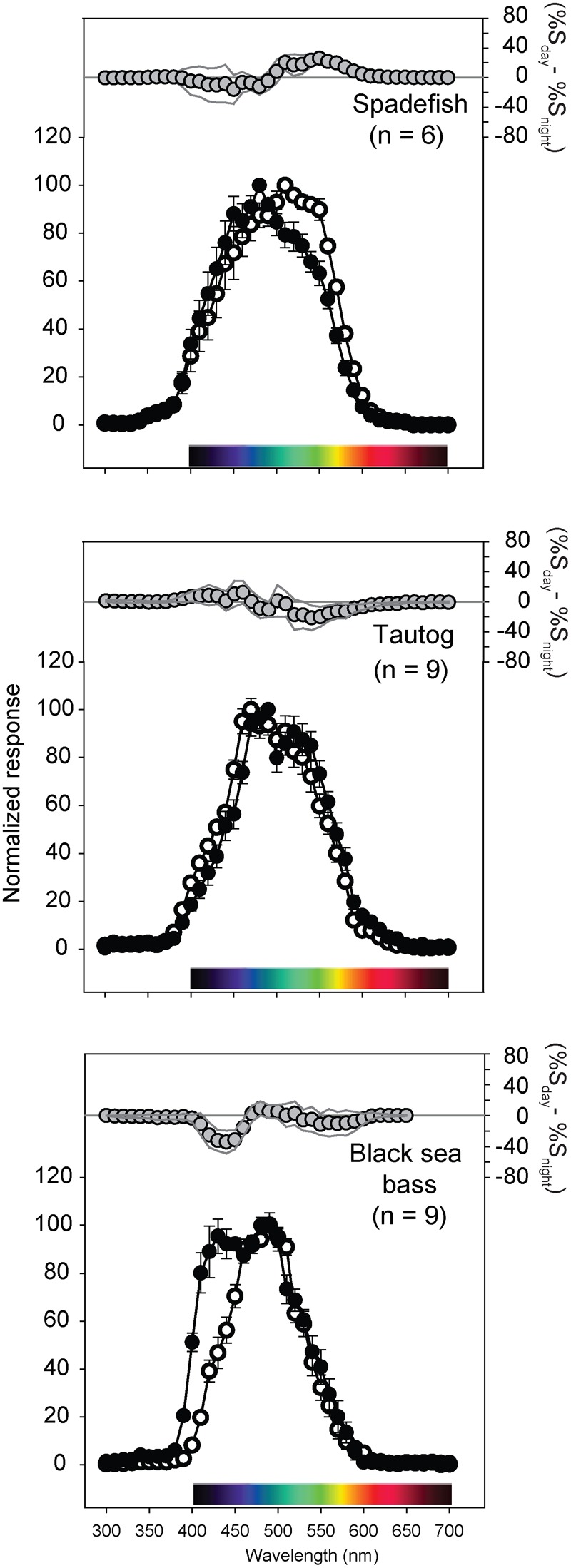
Spectral sensitivity curves and diel confidence intervals calculated from the electroretinograms (ERGs) of Atlantic spadefish, tautog, and black sea bass for wavelengths of 300–700 nm. Each species' curve is an average of six to nine individuals. Responses at each wavelength were normalised to the wavelength of maximal voltage response (V_max_) for each individual. Open symbols represent day experiments, filled symbols represent night experiments. Error bars are ± 1 SE. For each species, the top panels (grey circles, right axes) are the diel differences in spectral electroretinograms (ERGs) calculated by subtracting the day spectral responses (R_day_) from night responses (R_night_). Thin grey lines are ± 95% CI, calculated as 1.96 (s.e.m.). Values above the horizontal zero line (i.e. positive) indicate wavelengths of greater response during daylight, those below the zero line (i.e. negative) indicate wavelengths of greater nocturnal response. Significant diel differences occurred when CI did not encompass zero.

**Fig. 5.**
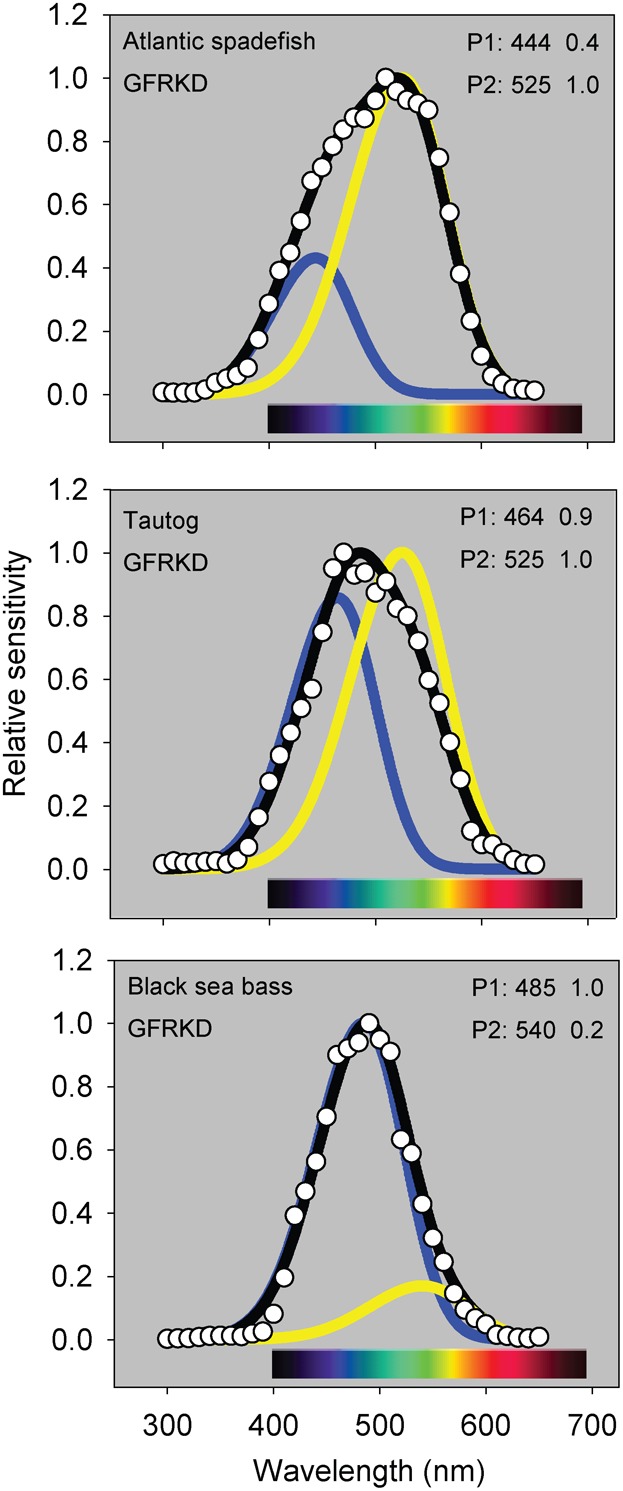
SSH (Stavenga et al., 1993) and GFRKD (Govardovskii et al., 2000) vitamin A1 templates fitted to day (photopic) temperate reef fish spectral ERG data by maximum likelihood (*sensu* Horodysky et al., 2008; Horodysky et al., 2010). Only estimates from best fitting models from Table 2 were plotted for each species. Values to the right of each pigment label are estimated λ_max_ and pigment specific weight as estimated by the model. P1 (blue or green line) is the short wavelength pigment, P2 (yellow or red line) is the intermediate or longer wavelength pigment. Black lines represent additive curves developed by summing the product of each curve weighted by the estimated weighting factor.

The photopic spectral sensitivities of the three temperate reef fishes generally spanned 400–600 nm, with black sea bass having the narrowest and most short-wavelength-shifted spectral range (Fig. 4). Atlantic spadefish and black sea bass demonstrated a significant nocturnal short wavelength shift, while tautog did not (Fig. 4). Maximum likelihood estimation using SSH and GFRKD rhodopsin templates suggested that the temperate reef fishes have multiple retinal pigments (Fig. 5). Spadefish (GFRKD; λ_max_ = 444, 525 nm), tautog (GFRKD; λ_max_ = 464, 525 nm) and black sea bass (GFRKD; λ_max_ = 485, 540 nm) photopic spectral sensitivities were consistent with the presence of at least two α-band vitamin A1 pigments (Table 2).

**Table 2.**
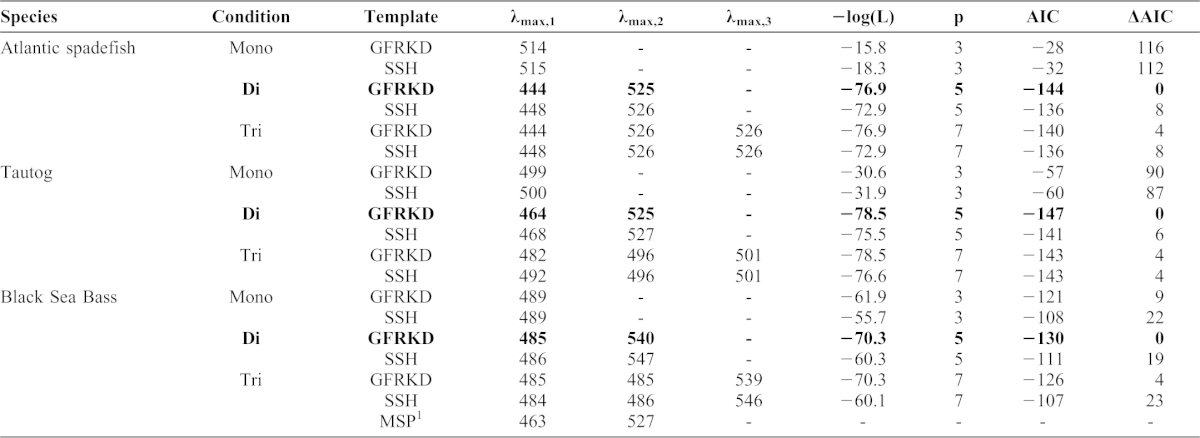
Parameter estimates and model rankings of SSH (Stavenga et al., 1993) and GFRKD (Govardovskii et al., 2000) vitamin A1 rhodopsin templates fitted to mid-Atlantic temperate reef fish spectral ERG data via maximum likelihood. The character “p” refers to the number of parameters in a model, “Mono”  =  monochromatic, “Di”  =  dichromatic, “Tri”  =  trichromatic. Only alpha bands of pigments were considered. The number below λ_max,1_ refers to pigment 1, etc. Bold type indicates the best supported pigment and template scenarios based on Akaike's Information Criterion (AIC) values (lower is better). ΔAIC is defined as the difference between the best fitting model and the models being compared (0–2  =  plausible, 2–4  =  reduced support, ≥10  =  no support). MSP  =  microspectrophotometry estimates of pigment λ_max,_ from the literature: ^1^Singarajah and Harosi, 1992.

## Discussion

Luminous sensitivities of temperate reef fishes, evidenced by the K_50_ points and dynamic ranges of V/logI curves, are comparable to other mid-Atlantic fishes (Horodysky et al., 2008; Horodysky et al., 2010) and a range of freshwater and marine teleosts (Naka and Rushton, 1966; Kaneko and Tachibana, 1985; Wang and Mangel, 1996; Brill et al., 2008). Mid-Atlantic temperate reef fishes demonstrated luminous sensitivities similar to coastal piscivores and benthic fishes (Horodysky et al., 2010), with less sensitivity than deep sea fishes (Warrant, 2000) and mesopelagic arthropods (Frank, 2003). Atlantic spadefish and tautog had similar K_50_ values (−0.1–0.24 log cd m^−2^) to estuarine sciaenids (0.2–0.3 cd m^−2^) and flatfishes (0.14–0.17 cd m^−2^), but fairly narrow dynamic ranges similar to those of coastal piscivores such as bluefish and cobia (Brill et al., 2008; Horodysky et al., 2008; Horodysky et al., 2010). In daylight, the luminous sensitivities of black sea bass were substantially more right-shifted (i.e., less sensitive), presumably as a result of retinomotor movements and migration of screened pigments (Ali, 1975); their high photopic K_50_ (∼0.74 cd m^−2^) and nocturnal increases in sensitivity of ∼0.75 log units are very similar to bluefish (*Pomatomus saltatrix* Linnaeus 1766; Horodysky et al., 2010). The luminous sensitivities of temperate reef fishes are thus at the more sensitive end of the continuum for coastal fishes, consistent with their use of less turbid but deeper and dimmer light habitats.

Temporal properties of temperate reef fish visual systems are also comparable to a range of diurnal freshwater and marine fishes, matching species-specific visual requirements and lifestyles (Table 3). The FFF of the three temperate reef fishes increased with light intensity (*sensu* Crozier et al., 1938), as has been observed in estuarine sciaenids and coastal piscivores (Horodysky et al., 2008; Horodysky et al., 2010). Collectively, maximum FFFs of temperate reef fishes were similar to benthic and nocturnal species in coastal and estuarine waters and lower than those of daytime foraging pelagic species. The highest photopic FFF_max_ of the schooling ephippid Atlantic spadefish (60 Hz) is comparable to coastal piscivores such as spotted seatrout (*Cynoscion nebulosus* Cuvier 1830) and cobia (*Rachycentron canadum* Linnaeus 1766; Horodysky et al., 2010). Serranid black sea bass, which orient in or above temperate reefs, had intermediate photopic FFF_max_ (52 Hz), similar to benthic summer flounder (*Paralichthys dentatus* Linnaeus 1766; 52 Hz) and turbid estuarine and coastal predators such as sandbar sharks (*Carcharhinus plumbeus* Nardo 1827; 54 Hz) and red drum (*Sciaenops ocellatus* Linnaeus 1766; 53 Hz; Table 3). The slower photopic FFF_max_ of the cryptic temperate labrid, tautog (48 Hz), is comparable to coastal sparids and lutjanids (McComb et al., 2013). Deeper-dwelling tautog and black sea bass had lower FFF at I_25_ than the more pelagic Atlantic spadefish, consistent with the presumably dimmer light niches of the former two species. The above metanalysis may be limited by differences in ecosystems as well as experimental and analytical techniques among these many studies; however, we consider the collective synthesis to be consistent with ecologies of the species discussed.

**Table 3.**
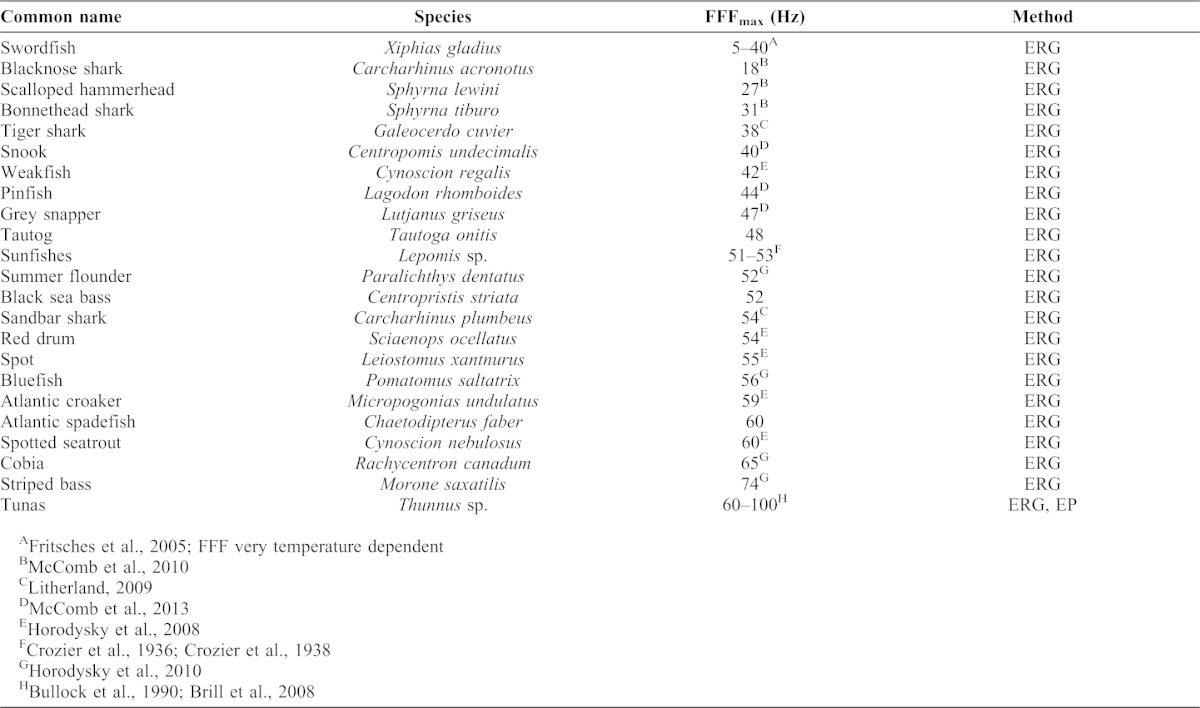
Maximum photopic temporal resolution (FFF_max_) of estuarine, coastal, and pelagic fishes. Methods of determination are electroretinography (ERG) or evoked potentials (EP). Data for Atlantic spadefish, tautog, and black sea bass are from this study.

Chromatic properties of the visual systems of Atlantic spadefish, tautog, and black sea bass can likewise be placed in context of fishes from coastal and other ecosystems. Coastal fishes are generally sensitive to a shorter subset of wavelengths than many freshwater fishes and a longer range of wavelengths than deep sea and oceanic species (Levine and MacNichol, 1979; Marshall et al., 2003a; Marshall et al., 2003b). This appears to be the case with the shallower-dwelling and more coastally-oriented Atlantic spadefish, which are comparatively more sensitive to slightly longer (green) wavelengths, whereas deeper-dwelling adult tautog and seabass are more sensitive to shorter (blue) wavelengths. Maximum sensitivity in an organism's light microhabitat is conveyed via scotopic (rod-based) pigment absorption spectra that match the ambient background to optimize photon capture (‘Sensitivity Hypothesis’: Bayliss et al., 1936; Clarke, 1936) whereas maximal contrast between an object and the visual background is provided by a combination of matched and offset visual pigments (‘Contrast Hypothesis’: Lythgoe, 1968). Fishes with multiple visual pigments likely use both mechanisms, depending on the phylogenetic, physical, and physiological constraints (McFarland and Munz, 1975). The three mid-Atlantic temperate reef fishes demonstrated broad, species-specific responses ranging from blue (∼440 nm) to green-yellow (570 nm) wavelengths (Fig. 4). Responses blue-shifted nocturnally in Atlantic spadefish and black sea bass, whereas tautog showed no diel shifts. Coastal and estuarine fishes are commonly dichromats possessing short wavelength visual pigments with λ_max_ values ranging from 440–460 nm and intermediate wavelength pigments with λ_max_ values of 520–540 nm (Lythgoe and Partridge, 1991; Lythgoe et al., 1994; Jokela-Määttä et al., 2007; Horodysky et al., 2008; Horodysky et al., 2010).

Chromatic sensitivities of the three temperate reef fishes were consistent with the presence of multiple pigments (Table 2). All three species appear to have at least two cone pigments: a rhodopsin sensitive to short blue wavelengths (440–480 nm) and one sensitive to intermediate green wavelengths (520–540 nm). Combining ERG with template fitting procedures is well-suited for comparative investigations of vision and form:function relationships in fishes (Brill et al., 2008; Horodysky et al., 2008; Horodysky et al., 2010; Matsumoto et al., 2012; McComb et al., 2013). Comparisons of MSP estimates to those resulting from the rhodopsin template fitting procedures applied to ERG data suggest that the latter provides useful comparative insights in visual systems with few, fairly widely spaced visual pigments (Horodysky et al., 2010). Published MSP data for small (presumably juvenile) black sea bass caught along a Massachusetts seawall suggested the presence of a 463 nm blue-sensitive and 527 nm green-sensitive pigment in the species (Table 2) (Singarajah and Harosi, 1992). Our λ_max_ estimates for larger ocean-caught adult females were shifted towards longer wavelengths but were also consistent with the presence of blue and green-sensitive pigments in the species. As suggested by Horodysky et al. (Horodysky et al., 2010), rhodopsin template fitting procedures may not extract the exact λ_max_ values from prior MSP studies due to potential differences in ontogenetic state and habitats of subjects, as a result of filtering by preretinal ocular media, experimental error in either ERG or MSP experiments, the generally poor performance of rhodopsin templates at short wavelengths (Govardovskii et al., 2000), or a combination of these factors. Electroretinography measures summed retinal potentials that account for any filtration by ocular media, which MSP does not (Brown, 1968; Ali and Muntz, 1975). Selective isolation of individual photopigments, chromatic adaptation, or behavioral experiments may help determine the presence of multiple cone mechanisms (Barry and Hawryshyn, 1999; Parkyn and Hawryshyn, 2000). However, cone morphologies, the specific photopigments they contain, and photoreceptor distributions were beyond the scope of our study. Collectively, the luminous, temporal, and chromatic properties of the visual systems of these three mid-Atlantic temperate reef fishes are consistent with inferences based on ecology and lifestyle.

**Fig. 6.**
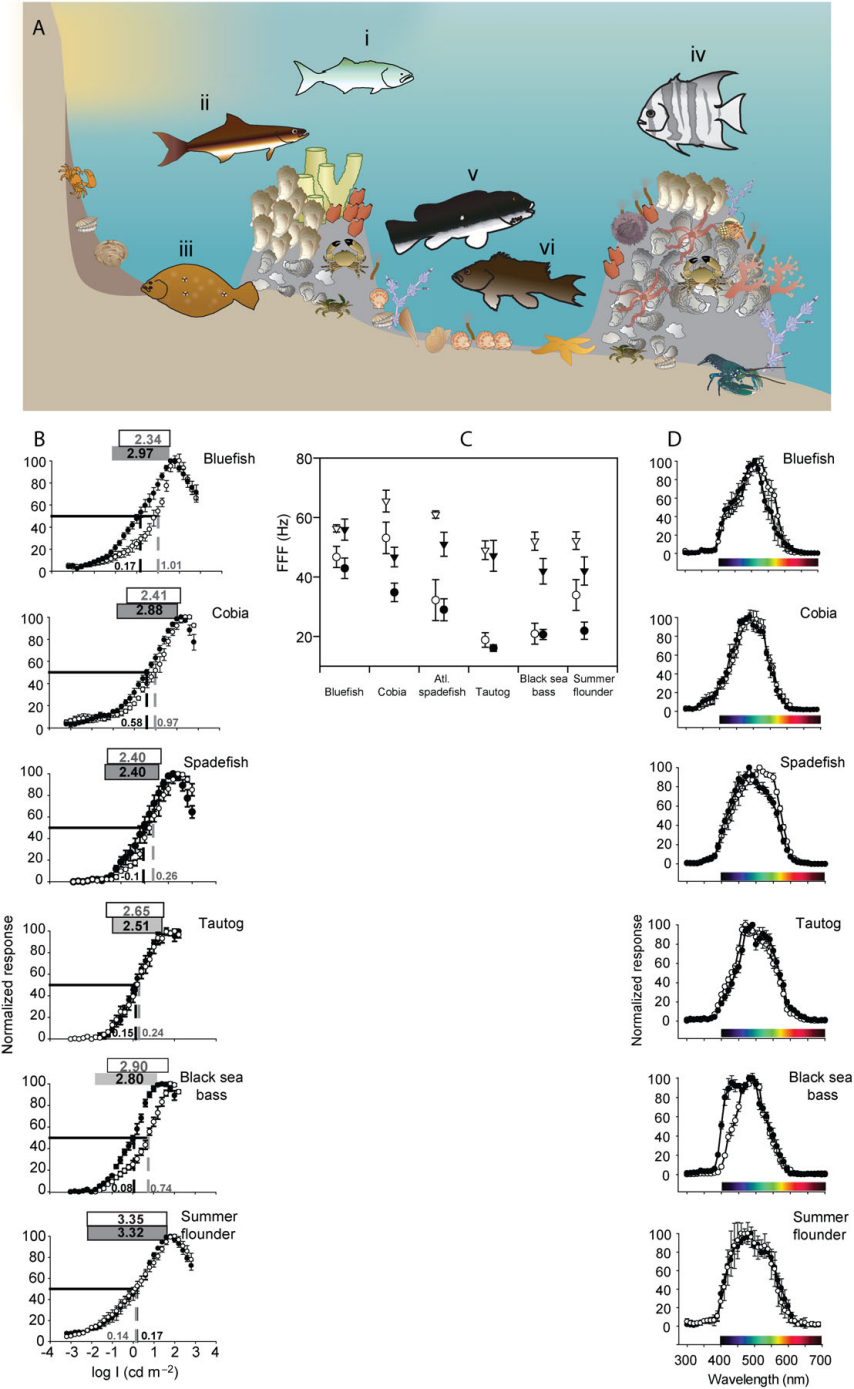
Comparative visual function of six mid-Atlantic predators that use temperate reefs and adjoining habitats. Data for bluefish (Ai), cobia (Aii), and summer flounder (Aiii) are from Horodysky et al. (Horodysky et al., 2010). Data for Atlantic spadefish (Aiv), tautog (Av), and black sea bass (Avi) are from the present study. For all panels, open symbols and grey text are the result of day experiments, closed symbols and black text are the result of night experiments. All error bars indicate ± 1 s.e.m. A. Conceptual diagram of the microhabitat specialization of the six temperate reef-associated fishes. B. Intensity-response electroretinograms (ERGs) of the six temperate reef-associated fishes. Each species' intensity-response curve is an average at least 5 individuals. Shaded boxes represent the dynamic range and breadth of each species in log candela m^−2^: photopic (white box, grey text), scotopic (dark grey, black text). Dashed vertical lines and adjacent numbers indicate K_50_ points. C. Mean flicker fusion frequency (FFF) values for the six temperate reef-associated fishes. Triangles are the FFF at maximum stimulus intensity (I_max_); circles are FFF at 25% of I_max_, considered to be a proxy for ambient environmental light intensity. D. Spectral sensitivity curves calculated from the ERGs of the six temperate reef-associated fishes for wavelengths of 300–700 nm. Responses at each wavelength were normalized to the wavelength of maximum response (V_max_) for each individual. Invertebrate symbols are courtesy of the Integration and Application Network, University of Maryland Center for Environmental Science (http://ian.umces.edu/symbols).

Reef-associated fishes show a wide range of visual properties and optical pigments depending on lifestyle and habitat, particularly in clear tropical habitats (Losey et al., 2003; Marshall et al., 2006). Temperate reefs, hard bottom habitats, and manmade structures of the mid-Atlantic region face less solar radiation, greener and more turbid waters, and larger annual temperature variation than tropical coral reef habitats (Steimle and Zetlin, 2000). Nonetheless, temperate reefs of the mid-Atlantic support numerous invertebrate and vertebrate fisheries and harbor many taxonomic representatives and ecological analogues of tropical coral reef fauna (Fig. 6).

Atlantic spadefish commonly school near and above manmade and natural reef and hardbottom habitats from New England to Brazil, where they feed on gelatinous zooplankton, hydroids, anthozoans, and amphipods and other epifaunal crustaceans (Hayse, 1990). There are no studies of the visual ecophysiology of other ephippid genera. However, numerous similar perciformes such as the rabbitfishes (Siganidae), moorish idols (Zanclidae) and surgeonfishes (Acanthuridae) have similar rhodopsin λ_max_ values in the 440 nm and 510–520 nm range (Losey et al., 2003). Spadefish coloration features vertical dark brown/black barring on a silver/white background, a common ‘dark/light adjacency’ strategy among reef fishes to maximize contrast against both pelagic water and optically complex reef backgrounds (Marshall et al., 2006). The alternating stripes may refer to the spatial frequency detecting capacity of a predator's retina, which may aid in camouflage under certain combinations of intensity and contrast against the background (Cott, 1939). The broadly-tuned dichromatic visual system and fairly fast temporal resolution of Atlantic spadefish is well suited to the optical properties of both inshore and offshore water columns used by this species in temperate mid-Atlantic waters.

Tautog exhibit sexual dimorphism and male territoriality, yet are not hermaphroditic like other labrids (Hobson, 1968; Hobson, 1972; Olla et al., 1974; White et al., 2003). Daily cycles of foraging activity in tautog and many other labrids are highly correlated to ambient light; tautog feed on sessile mollusks and small crustaceans during daylight hours before returning to nocturnal refugia in natural reefs and rock outcroppings as well as man-made structures such as jetties, bridge-tunnel networks, artificial reefs, and shipwrecks (Olla et al., 1974; White et al., 2003). Tautog range from Nova Scotia to South Carolina and undertake both ontogenetic and seasonal inshore-offshore movements induced by temperature (Olla et al., 1974; Auster, 1989; Arendt et al., 2001). Coloration is sex-specific in the species, with a more cryptic mottled brown coloration in juveniles and females whereas males are conspicuously colored in near solid black punctuated by a lateral white spot and underlain by a bright white ventrum (Auster, 1989). As such, the coloration of juveniles and females may primarily be for camouflage, whereas the conspicuous, high-contrast coloration of adult males may enhance territorial defense and attract mates (Olla et al., 1981), as has been shown for other wrasses (Barry and Hawryshyn, 1999; Marshall, 2000). The dichromatic visual system of tautog is thus well suited to the optical properties of both inshore and offshore reef and hardbottom habitats, and the diel invariance of temporal resolution as well as luminous and spectral sensitivity in the species is in line with their nocturnal torpor.

Black sea bass are incompletely metagonous, structure-associated protogynous hermaphrodites that are predators of a myriad of mobile crustaceans, bivalves, and small to medium-sized fishes in temperate reefs from Nova Scotia to Florida (Musick and Mercer, 1977; Sedberry, 1988; Steimle and Figley, 1996). Black sea bass undertake both ontogenetic and seasonal inshore-offshore movements induced by temperature (Musick and Mercer, 1977; Mercer, 1979). As in many territorial protogynous serranids, size and coloration in black sea bass is dimorphic, featuring a more cryptic mottled brown coloration in juveniles and females whereas males have a brilliant blue adipose nuccal hump (Lavenda, 1949; Murdy and Musick, 2013). As with tautog, the coloration of juveniles and females may primarily be for camouflage, whereas the conspicuous, high-contrast blue and mottled brown, black, and white display of adult males may enhance territorial defense and attract mates (Olla et al., 1981), as has been shown for other reef fishes (Barry and Hawryshyn, 1999; Marshall, 2000). Collectively, the dichromatic visual system of black sea bass is thus well suited to the optical properties of both inshore and offshore reef and hardbottom habitats, and the diel increases in sensitivity and nocturnal blue-shift may extend the visual foraging of the species into crepuscular periods.

Optical conditions in coastal waters are complex and have changed dramatically over the past century due to human activities (Kemp et al., 2005), with potentially large consequences for visually-foraging fishes (Aksnes, 2007; Horodysky et al., 2010). Increasing turbidity affects the distances over which temperate reef fishes can communicate with conspecifics, discern predators, and locate prey. While optical conditions in mid-Atlantic temperate reefs are unlikely to be affected as dramatically as nearby estuarine waters by processes such as eutrophication and pollution, many fishes that associate with temperate reefs depend on visual coloration and displays for reproductive signaling (as with cichlids; Seehausen et al., 1997) in already dim and complex optical backgrounds. Describing the visual performance of temperate reef fishes is a first step, but a better understanding is required of ambient light levels in specific light niches (Marshall et al., 2006), light threshold effects on foraging and predator-prey interactions (Mazur and Beauchamp, 2006; De Robertis et al., 2003), reproductive signaling and reproduction (Engström-Östa and Candolin, 2007), as well as interactions of these three fisheries resources with fishing gear (Buijse et al., 1992). Similarly, the effects of ambient light fields on the reflectance of conspecifics (especially during nuptial and agonistic displays) and prey, and the manner in which these change in space and time should also be investigated to gain insights into visual systems and tasks for these species (Levine and MacNichol, 1979; Johnsen, 2002). Comparative approaches investigating the form-function-environment relationships between sensory ecophysiology, behavioral ecology, and population processes are thus important for mechanistic understanding across scales from cells to populations to support better management of aquatic resources (Horodysky et al., 2010).
